# Association of anti-thyroid autoantibodies with neuropsychiatric features in patients with affective and schizophrenia spectrum disorders

**DOI:** 10.1192/j.eurpsy.2024.582

**Published:** 2024-08-27

**Authors:** R. S. Ilhan, K. C. Can, S. N. Lalic, E. Halaman, O. Aktay, F. Özdemir, B. Çolak, B. Duman, S. Yazıcı, M. C. Saka

**Affiliations:** ^1^Psychiatry, Ankara University, ANKARA, Türkiye; ^2^Psychiatry, Specil Psychiatric Hospital Department of Psychiatry Podvrsanska, psychiatry, Vrsac, Serbia; ^3^Psychiatry, mamak devlet hastanesi, ANKARA, Türkiye

## Abstract

**Introduction:**

A growing body of evidence has shown the association between autoimmune thyroiditis and mental illness (Rege *et al.* AUS N J S Psychiatry 2013; 300 141-154). Identifying the neuropsychiatric features associated with thyroid antibody positivity could have significant implications for diagnostic and therapeutic strategies. However, the link between anti-thyroid antibodies and precise underlying pathophysiology requires future research.

**Objectives:**

The aim of the present study was to conduct a retrospective evolution in patients diagnosed with schizophrenia spectrum disorder and affective disorder who were screened for anti-thyroid antibodies at the time of their hospitalization and to investigate neuropsychiatric features of anti-thyroid antibody-positive patients.

**Methods:**

A total of 143  inpatients diagnosed with schizophrenia spectrum disorders and affective disorders between 2021 and 2023 were screened for anti-thyroid antibodies such as thyroid peroxidase (TPO) and thyroglobulin (TG). All patients were women. In order to elucidate the subsequent neuropsychiatric clinical features of individuals with positive anti-thyroid antibodies, the retrospective examination was conducted based on Neuropsychiatric Invetory-Q (NPI-Q) and DSM-V diagnostic criteria utilized at the time of hospitalization.

**Results:**

The main age of the patients was 48.2 (SD 10.4). A total of 143 inpatients with schizophrenia spectrum disorders and affective disorders were screened for anti-thyroid antibodies at the time of their hospitalizations. %23.1 (n=33) tested positive for at least one of the anti-TG or anti-TPO. All patients were euthyroid. The neuropsychiatric diagnoses are shown in Table 1. The most common neuropsychiatric features assessed by NPI-Q are shown in Table 2. 12.1% (n=4) of all patients were treated with IV steroid Pulse therapy.

**Table 1.** Neuropsychiatric syndrom-level diagnostic patterns according to DSM-V

	Patients with positive thyroid autoantibodies (n=33)
Manic syndrome	10 (30.3%)
Psychotic Syndrome	19 ( 57.6%)
Depression syndrome	5 (15.2%)
Catatonia	10 (30.3%)
Exited	6 (18.2%)
Stuporus	2 (6.1%)
Fluctuating	2 (6.1%)

**Table 2.** The most common clusters of Neuropsychiatric features

NPI-Q	Positive Thyroid Autoantibodies (n=33)
Delusion	15 (45.4%)
Agitation/Aggression	14 (42.4%)
Irrıtability	14 (42.4%)
Motor abnormality	14 (42.4%)
Sleep disorder	15 (45.4%)
Appetite/Eating	14 (42.4%)

**Conclusions:**

In particular, in a subset of schizophrenia spectrum disorder or affective disorder patients with positive anti-thyroid antibodies may indicate autoimmunity, especially in cases where catatonic symptoms dominate the clinical presentation.

**Disclosure of Interest:**

None Declared

